# Characterization of a new mouse line triggering transient oligodendrocyte progenitor depletion

**DOI:** 10.1038/s41598-023-48926-4

**Published:** 2023-12-11

**Authors:** B. Brousse, O. Mercier, K. Magalon, P. Gubellini, P. Malapert, M. Cayre, P. Durbec

**Affiliations:** 1 Aix Marseille Univ, CNRS, IBDM UMR7288, Case 907, Parc Scientifique de Luminy, 13288 Marseille Cedex 09, France; 2 Aix Marseille Univ, CNRS, LNC UMR7291, 3 Place Victor Hugo, 13331, Marseille Cedex 3, France

**Keywords:** Oligodendrocyte, Mouse

## Abstract

Oligodendrocyte progenitor cells (OPC) are the main proliferative cells in the healthy adult brain. They produce new myelinating oligodendrocytes to ensure physiological myelin remodeling and regeneration after various pathological insults. Growing evidence suggests that OPC have other functions. Here, we aimed to develop an experimental model that allows the specific ablation of OPC at the adult stage to unravel possible new functions. We generated a transgenic mouse expressing a floxed human diphtheria toxin receptor under the control of the PDGFRa promoter, crossed with an Olig2Cre mouse to limit the recombination to the oligodendrocyte lineage in the central nervous system. We determined a diphtheria toxin dose to substantially decrease OPC density in the cortex and the corpus callosum without triggering side toxicity after a few daily injections. OPC density was normalized 7 days post-treatment, showing high repopulation capacity from few surviving OPC. We took advantage of this strong but transient depletion to show that OPC loss was associated with behavioral impairment, which was restored by OPC recovery, as well as disruption of the excitation/inhibition balance in the sensorimotor cortex, reinforcing the hypothesis of a neuromodulatory role of OPC in the adult brain.

## Introduction

Oligodendrogenesis starts during embryonic development with the production of successive waves of OPC from different ventro-dorsal domains of the neuroepithelium; these OPC will populate the whole central nervous system (CNS) (for review, see^1^). As they migrate, OPC continually adjust their trajectories to avoid other OPC by extending and retracting their processes, resulting in an even, nonoverlapping distribution of cells^2,3^. After birth, OPC pursue their differentiation program and generate myelinating oligodendrocytes (OLG). Myelination will continue for weeks (in mice) or years (in humans); it is thus the latest event in CNS development. A fraction of OPC does not differentiate into myelinating OLG but remains as quiescent progenitors in the adult brain, representing 2 to 9% of all neural cells^4^. Compared to their developmental counterpart, adult OPC exhibit lower proliferation and migration potential and are less responsive to growth factors^5–7^.

Despite their substantial abundance, the role of OPC in the adult brain has long been neglected, and studies addressing their function are relatively recent. The first significant discovery was their contribution to myelin repair. OPC are the main cell population able to produce new myelinating OLG after a demyelination insult^8–11^, but also after a trauma^12^ or in neurodegenerative disorders such as Alzheimer’s disease^13^. OPC also contribute to the formation and resolution of the glial scar after an injury^14^. In physiological conditions, OPC contribute to myelin remodeling: by symmetric and asymmetric divisions they self-renew and produce a new myelinating OLG^15,16^, leading to de novo myelination of naked axons or intercalation of new myelin segments. Interestingly, such myelin remodeling in the adult brain is activity-driven^17,18^, and preventing OPC from differentiating in healthy adult mice leads to impaired motor and cognitive learning^19–22^. Nevertheless, the physiological OLG turnover seems to be quite low, both in mice and humans^23,24^. The generation of new myelin sheaths has been recently observed from mature OLG using in vivo two-photon imaging^25^.

Twenty years ago, Bergles and Jahr^26^ discovered the presence of functional synaptic contacts between neurons and OPC and provided evidence for excitatory^26^ and inhibitory^27^ synaptic inputs onto OPC. These synapses are present throughout the brain in the gray^26,28–30^ and the white matter^31,32^. Synaptic activity between neurons and OPC influences OPC proliferation and differentiation into mature OLG (for review, see^33^), and is important for myelination and remyelination (for review, see^34^). An increasing number of pieces of evidence suggests that OPC cover a variety of functions independently of their canonical role in myelination. For instance, during neuroinflammation, OPC can be co-opted by immune cells and participate in antigen presentation^3^. During embryonic development, OPC contribute to neuronal circuit refinement via arbor growth regulation^35^, axonal pruning and synapse engulfment^36,37^. They also control cortical interneuron migration^38^ and can modulate the surrounding neuronal network via activity-dependent processing of NG2^39^. Interestingly, some OPC express voltage-gated sodium and potassium channels, allowing them to generate action potentials^40^. Furthermore, using optogenetic techniques, recent work in the hippocampus has shown that OPC can release GABA on proximal GABAergic interneurons, tuning inhibitory synaptic activity^41^. Given their electrophysiological properties and ability to communicate with excitatory and inhibitory neurons, OPC are thus ideally positioned to endorse neuromodulatory functions.

Few genetically modified mouse models have been generated to delete the OPC population to unravel the new functions of these cells. One is the NG2Cre:DTR^flx/flx^ mouse line, in which diphtheria toxin (DT) injections trigger OPC death^42^. However, this is not specific since pericytes also express NG2 and these cells play important roles in brain homeostasis, including in myelin integrity^43^. Another model is the Sox10-iCreERT2:Esco2^flx/flx^ mouse line^44^; in this case, after tamoxifen-induced recombination, only proliferating OPC will be targeted and die, leading to a severe reduction of new OLG production in the adult brain. Moreover, in this model, the number of OPC remained stable due to the slow elimination of recombined cells and the enhanced proliferation of non-recombined cells. Merson’s lab recently obtained efficient and lasting OPC ablation using a pharmacogenetic approach targeting both OPC and SVZ-derived neural progenitors^45^. Here, we aimed to generate a new mouse model to thoroughly kill the population of OPC in the adult CNS in a very specific and time-controlled manner, based on the targeted expression of the human DT receptor (DTR). We produced a knock-in mouse by inserting a LoxP-Venus-LoxP-DTR cassette in the PDGFRa locus. Since PDGFRa is expressed at a low level in pericytes^46^ and in stromal cells^47^, we ensured specificity by crossing these PDGFRa-VenusStopFlox-DTR mice with Olig2Cre mice. We thus obtained Olig2Cre:PDGFRaDTR mice in which DTR is specifically expressed in OPC. We fully characterized OPC depletion in this model in the white (corpus callosum (CC)) and the grey (cortex (CX)) matter following different DT injection protocols. We show that this model allows producing a significant but transient reduction in OPC density in both the CC and the CX without nonspecific toxicity, which makes it a unique tool to study OPC function independently of OLG and myelin production. Interestingly, the decrease in OPC density was associated with some behavioral defects and reduced excitatory input on layer 5 (L5) cortical projection neurons (CPNs), supporting the physiological role of OPC in regulating information processing in the adult brain.

## Results

### Generation of a new mouse line with temporally controlled DTR expression in OPC

In order to specifically ablate OPC, we generated a knock-in mouse that expresses the human DTR under the transcriptomic control of PDGFRa. A plasmid with the following cassette was produced: PDGFRa5’arm-loxP-Venus-pA-loxP-DTR-pA-FRT-Neo-FRT-PDGFRa3’arm (total cassette length 13.4 kb; see “Methods”) (Supplementary Fig. S1a). The construct was electroporated in mouse ES cells for homologous recombination (SEAT Service des Animaux Transgéniques-Villejuif). Among 220 ES clones tested by PCR and southern blot, only one (clone 4C10) had indeed properly recombined in the PDGFRa locus (Supplementary Fig. S1b). This clone was microinjected in blastocysts and 4 chimeras were obtained, among which only one male was able to reproduce with a wild-type female and to transmit the transgene to one female (female 1.23). This female was thus the founder of the colony. The FRT sequence was eliminated by crossing the founder progeny with an actin-Flipase male. We thus obtained a new mouse line (PDGFRa-VenusStopFlox-DTR) in which DTR expression is under the control of PDGFRa promoter but is dependent on Cre-mediated removal of transcriptional STOP cassette (Supplementary Fig. S1a).

**Figure 1:**
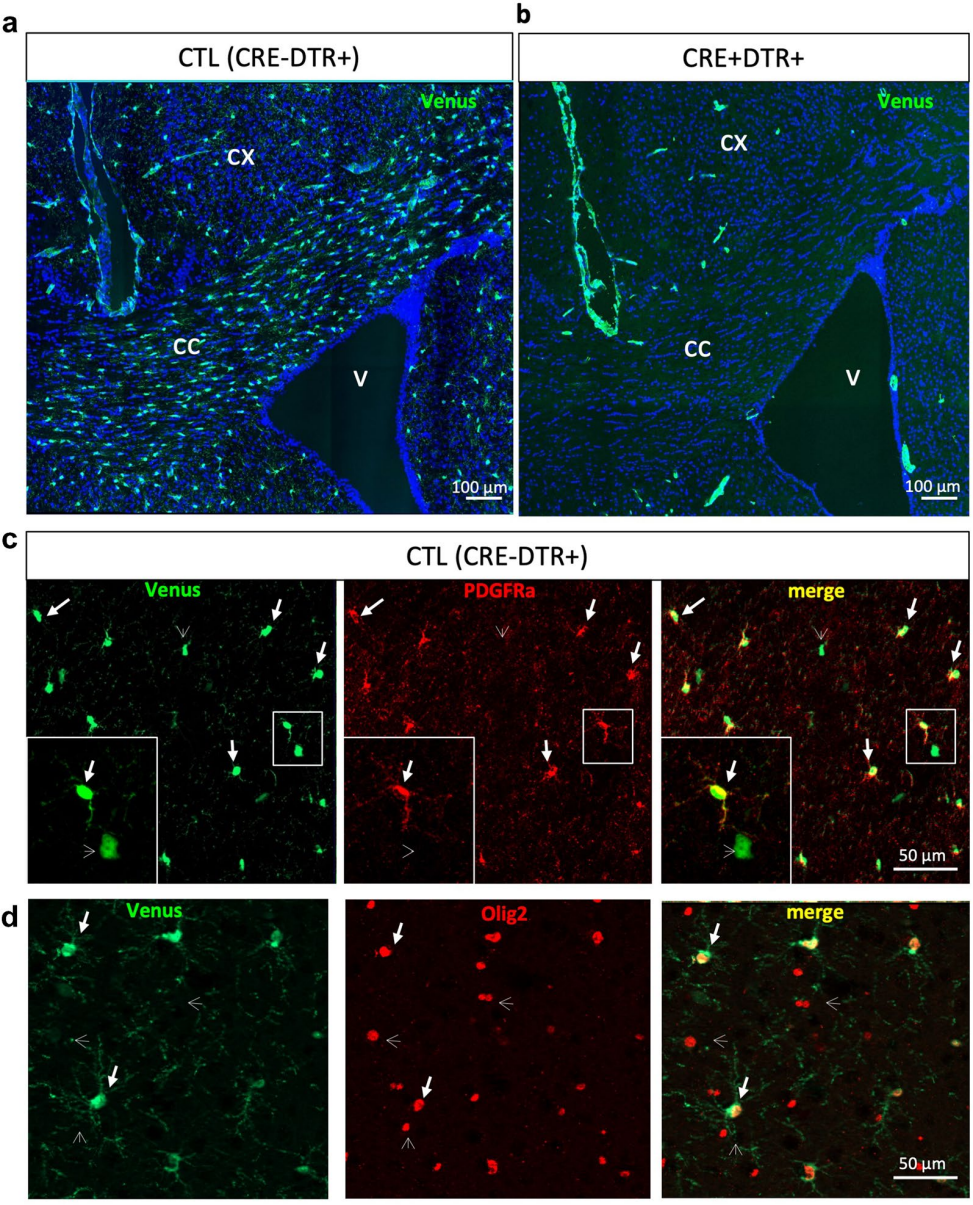
Transgene expression is specific to OPC. Venus expression in absence (**a**) or presence (**b**) of Cre recombinase in cells of the oligodendrocyte lineage (Olig2 + cells). The numerous and widespread Venus + cells observed in CTL (CRE-DTR +) mice (**a**) are not present in CRE + DTR + mice (**b**), attesting the efficacy of excision of the Venus-Stop cassette by the Cre recombinase. (**c**) Double immunolabeling of Venus and PDGFRa in CTL (CRE-DTR +) mice. All PDGFRa + cells are also Venus + (arrows), but few Venus + cells are not PDGFRa (arrowheads). The small white square with one double positive cell and one cell Venus + PDGFRa- is shown enlarged in the lower left corner. (**d**) double immunolabeling of Venus and Olig2 in CTL (CRE-DTR +) mice. Only a fraction of Olig2 + cells are Venus + ; by contrast, all Venus + cells are Olig2 + (arrows), suggesting that only OPC express Venus. CX: cortex ; CC: corpus callosum ; V: ventricle.

Histological brain sections of these mice show numerous Venus + cells, with particularly high density in the CC (Fig. 1a). As expected, Venus labeling disappears when mice were crossed with Olig2CRE mice (Fig. 1b): in the CRE + DTR + progeny, only meninges and some blood vessels remain labeled. This is consistent with PDGFRa expression in pericytes, but since these cells do not express Olig2, the Venus cassette is not excised, thus they remain labeled in CRE + DTR + mice.

We checked the specificity of Venus expression by immunofluorescence and observed a very good correspondence between Venus and PDGFRa labeling (Fig. 1c). Aside from blood vessels, almost all Venus + cells (98%) were also Olig2 + in the CC and in the CX, demonstrating their belonging to the oligodendrocyte lineage (Fig. 1d, Supplementary Table S1). Some Venus + cells were PDGFRa-; they can be assumed to be differentiating OPC that lost PDGFRa expression but remained Venus + due to slower protein degradation. Indeed, 13 to 18% of Venus + cells expressed CC1 (Supplementary Table S1).

**Figure 2:**
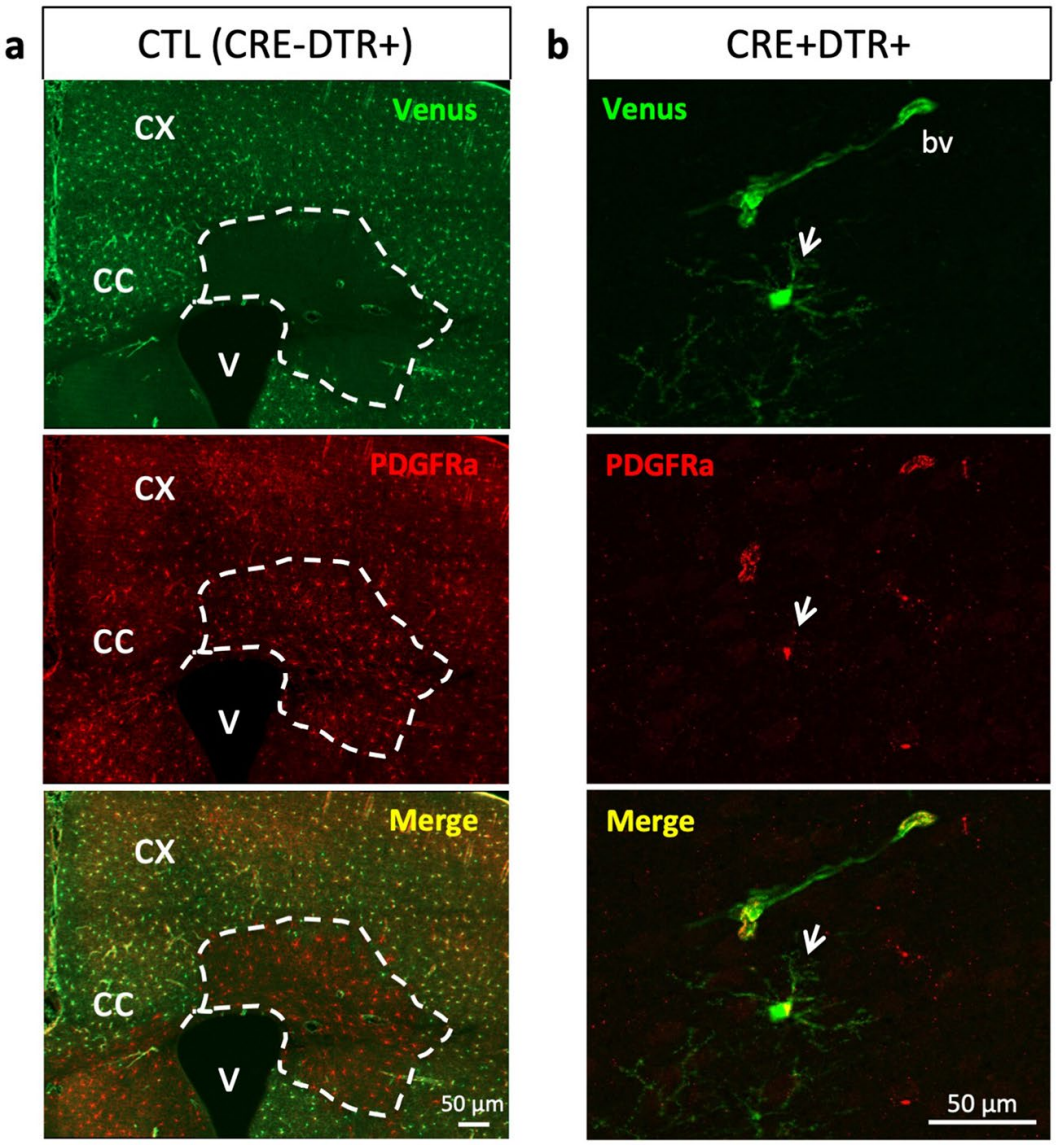
Presence of patches suggesting loss of transgene and recombination failure in few OPC. (**a**) Illustration of a patch devoid of Venus expression in a CTL (CRE-DTR +) mouse, despite the presence of PDGFRa + cells. (**b**) Illustration of an isolated Venus + PDGFRa + cell (arrow) in the CX of CRE + DTR + mouse, shown at higher magnification on the right side. CX: cortex; CC: corpus callosum ; V: ventricle ; bv: blood vessel.

In some animals we observed few patches devoid of Venus + cells (Fig. 2a, Supplementary Fig. S2), suggesting that some OPC could have lost or repressed the transgene during development, and by proliferation they have generated clones of cells devoid of Venus expression. Conversely, after crossing with Olig2CRE mice, we observed sparse isolated Venus + OPC (Fig. 2b), indicating that recombination could have failed in very few cells.

### Depletion of OPC in corpus callosum and cortex after diphtheria toxin injection

**Figure 3:**
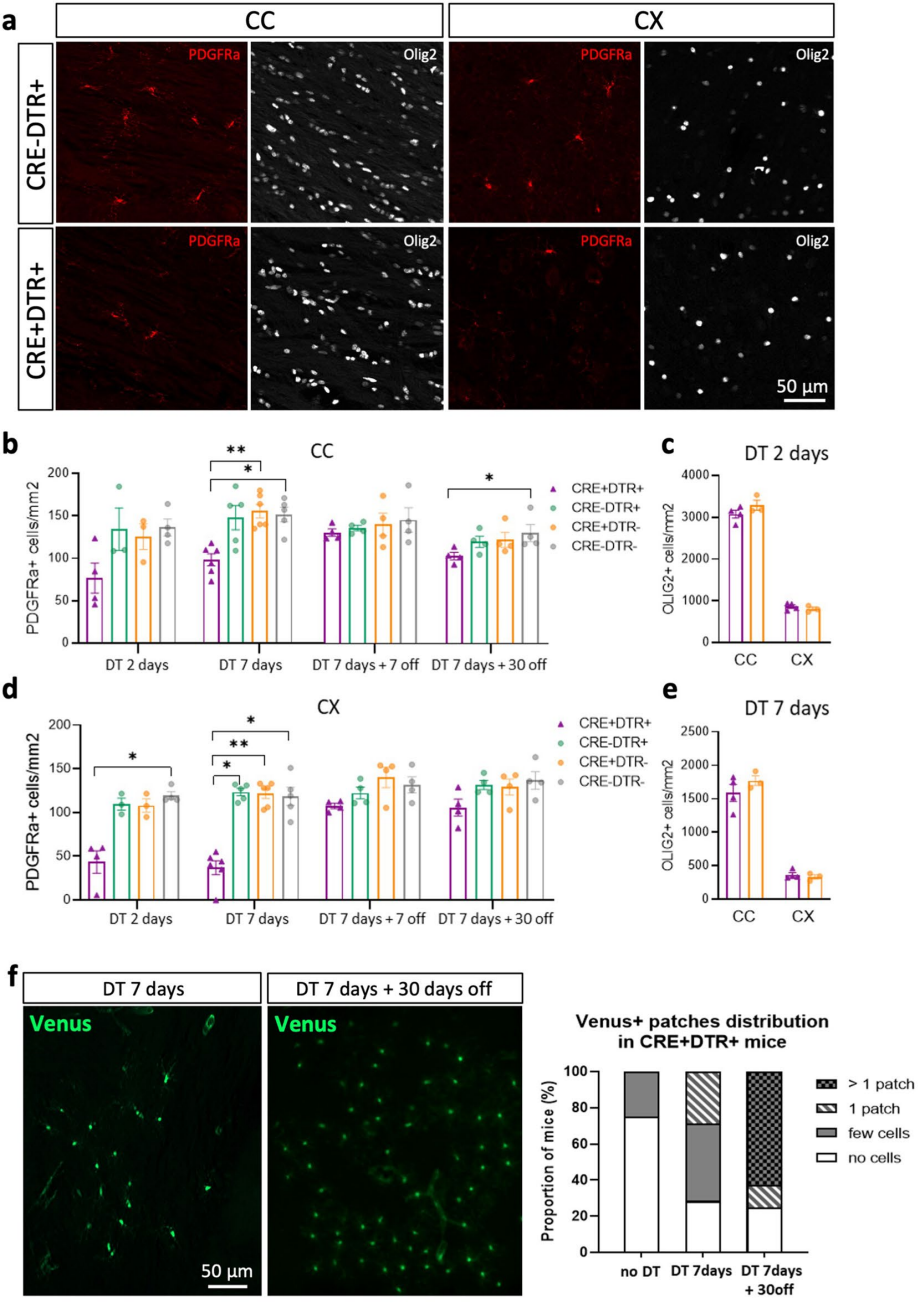
Histological characterization of OPC depletion and recovery. (**a**) Illustration of PDGFRa and Olig2 + immunolabelling in CC and CX after one-week daily SC injections of DT. CC: corpus callosum, CX: cortex. (**b**) 2 and 7 days of daily injection at 20 ng/g in the CC, followed by recovery after 7 and 30 days. (**c**) 2 days of daily injection at 20 ng/g does not affect Olig2 + cell density in CC and CX (**d**). 2 and 7 days of daily injection at 20 ng/g in the CX, followed by recovery after 7 and 30 days. (**e**) 7 days of daily injection at 20 ng/g does not affect Olig2 + cell density in CC and CX. Results are expressed as mean ± SEM (dots = single animal value in each group; Mann–Whitney test; *p ≤ 0.05; Kruskal–Wallis test followed by Dunn’s multiple comparisons test; *p ≤ 0.05; **p ≤ 0.01). (**f**) Illustration and quantification of Venus + patches in mice sacrificed immediately (left picture) or 1 month (right picture) after the end of 7 daily DT injections. Note the enlarged size of the patch and the increased probability to observe patches in mice after 1-month delay (Chi-square test).

We then characterized the efficiency of OPC depletion after DT injections in the adult mouse brain. Several doses of DT were tested and injected daily during one week by intraperitoneal (IP) route. Animals were killed 1 day after the last DT injection. OPC density was quantified based on PDGFRa immunolabeling and the oligodendrocyte lineage with Olig2 immunolabeling. We established 20 ng/g as the optimal dose of DT able to trigger efficient OPC depletion without obvious toxic side effects. Indeed, the dose of 10 ng/g induced non significant reduction of OPC density in the CC (13%, *p* = 0.4) and mild reduction in the CX (35%, *p* = 0.02) of CRE + DTR + mice compared to control (CTL) mice (Supplementary Fig. S3a). In contrast, 40 ng/g daily injections led to weight loss and mortality (not shown). With the 20 ng/g DT dose, OPC density was significantly reduced in CRE + DTR + in both structures (CC: *p* = 0.007; CX: *p* = 0.008) (Fig. 3b,d), corresponding to a 36% ablation in CC (*p* = 0.0001) and 70% in CX (*p* < 0.0001) compared to all CTL mice.

Interestingly, similar results were obtained using subcutaneous (SC) injections, with up to 39% and 63% OPC density reduction in CC and CX, respectively (CC: 57 ± 5 vs. 93 ± 4 PDGFRa + cells/mm^2^, *p* = 0.0159 PDGFRa + cells/mm^2^; CX: 29 ± 9 vs. 80 ± 3 PDGFRa + cells/mm^2^, *p* = 0.0159; Supplementary Fig. S3b). Since this route is less invasive than IP injections, we preferred this protocol for functional experiments.

To assess the early effect of DT, we sacrificed mice after only 2 days of SC injections at 20 ng/g. At this time point, we observed 42% OPC depletion in the CC and 62% in the CX compared to pooled CTL (CC: 77 ± 18 vs. 133 ± 9 PDGFRa + cells/mm^2^, *p* = 0.024; CX: 44 ± 13 vs. 113 ± 4 PDGFRa + cells/mm^2^, *p* = 0.002). Despite a clear reduction of OPC density in the CC of CRE + DTR + mice, comparison with the three CTL groups showed no significant difference at this time point (*p* = 0.1482). In contrast, the decrease was already significant in the CX (*p* = 0.0091) (Fig. 3b,d).

Since Olig2-Cre mouse line is haploinsufficient for Olig2^48^, we characterized the density of Olig2 cells in Cre + DTR + compared with CRE + DTR- mice (both presenting the same number of copies of Olig2 gene). Olig2 cell density did not differ between CRE + DTR- and CRE + DTR + mice, suggesting that mature OLG were not affected by DT injection in our mouse model (Fig. 3c,e).

We also checked for glial reactivity following 2 or 7 days of DT injection using CD68 as a marker of activated microglia and GFAP for astrocytes. We observed very few CD68 + cells (Supplementary Fig. S4a, c) and astrocytes did not exhibit morphological changes or increased GFAP expression (Supplementary Fig. S4b, c). Quantitative analyses confirmed the lack of activation of either microglia or astrocytes in both structures (Supplementary Fig. S4d).

To sum up, one-week daily DT injections (20 ng/g) triggered efficient and selective OPC depletion in the adult mice brain without inducing a visible inflammatory response nor affecting animals’ health.

### OPC depletion is transient, followed by rapid recovery

We then examined if the OPC density drop was maintained in the long-term. We used the same protocol as previously (daily injection of 20 ng/g DT for 7 consecutive days) but waited 7 or 30 days after the last DT injection before histological analyses. 7 days after DT injections, OPC density was back to normal levels in the CC (*p* = 0.7518) and the CX (*p* = 0.2156) (Fig. 3b,d). After 30 days, although a minor difference between CTL groups and CRE + DTR + mice was observed in the CC (CC: *p* = 0.0313; Fig. 3b,d), we observed a restoration of OPC population in the CX (*p* = 0.1360; Fig. 3b,d).

We next questioned whether increasing the DT treatment duration can prolong OPC depletion. None of the protocols tested in which DT exposure was maintained for up to 5 weeks efficiently sustained OPC depletion (see Supplementary Fig. S5). These results indicate that sustained DT treatment for several weeks does not allow the maintenance of the OPC depletion observed after the one-week treatment.

We also observed few patches of Venus + cells in CRE + DTR + animals treated with DT for one week (Fig. 3f). The percentage of CRE + DTR + mice showing such patches and the size of the patches significantly increased with the delay between the end of DT injections and the sacrifice (*p* = 0.0069) (Fig. 3f). At 7 days of DT treatment, around 30% of mice exhibited 1 small patch of Venus + cells and none had more than 1. In contrast, more than 60% of mice killed 30 days after DT injections exhibited more than 1 big patch of Venus + cells (Fig. 3f). Since we did not observe any patches in non-injected mice (only 1–2 isolated cells in few sections), it can be suggested that these patches of Venus + cells are generated by the proliferation of sparse OPC that have escaped from the Cre recombination.

Altogether, these results show that OPC have a high recovery capacity and that a few spared cells can repopulate the brain parenchyma, which might account for the transient character of OPC depletion.

### Mice with acute OPC depletion show decreased locomotor activity and altered spatial working memory

**Figure 4:**
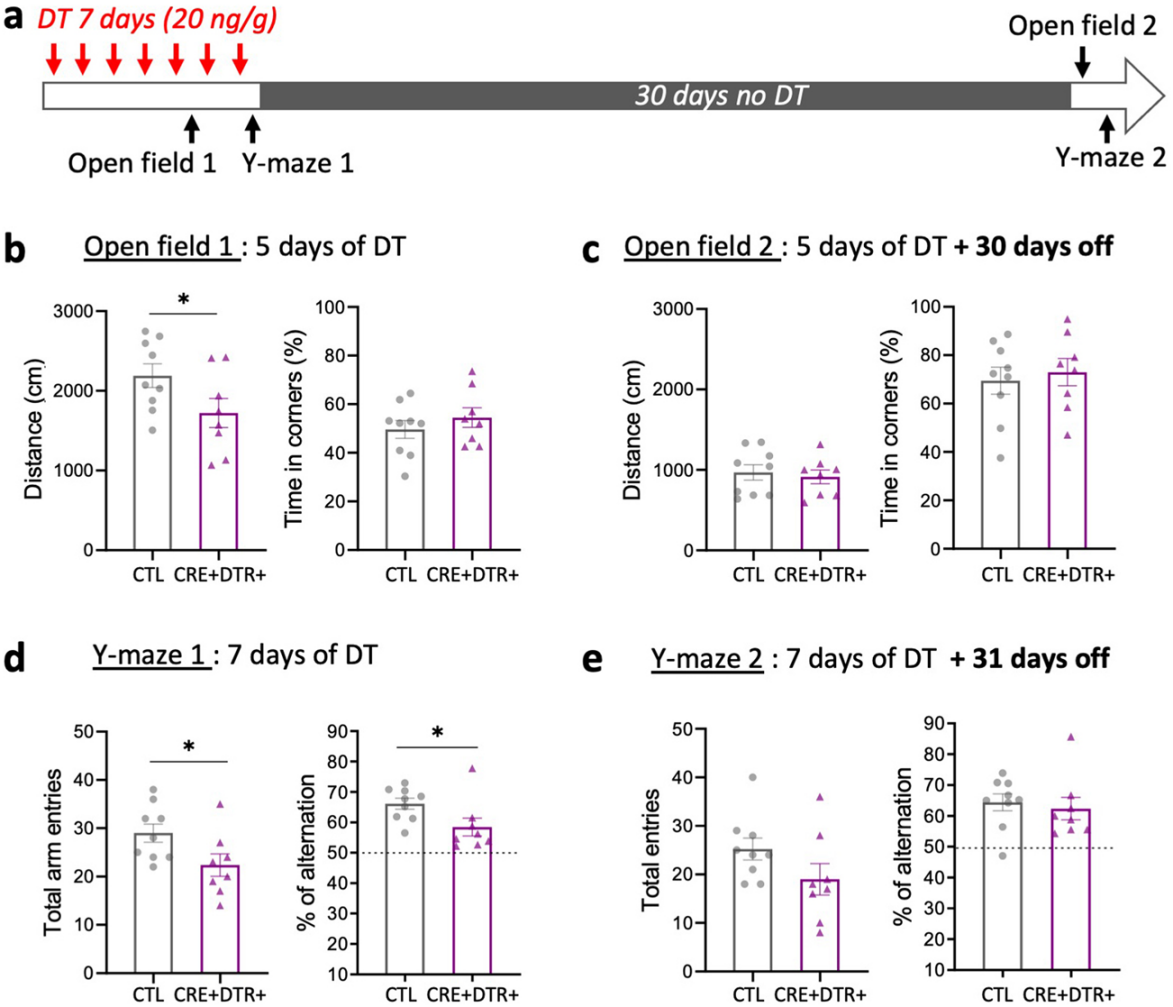
Mice with acute OPC ablation exhibit reduced locomotor activity and altered spatial working memory. (**a**) Timeline of behavioral tests. Mice were injected daily with DT (20 ng/g SC) for 7 days. The open field test was performed after 5 days of DT injections and the Y-maze test after 7 days of DT injections. The open field and the Y-maze tests were repeated 30 and 31 days after the end of DT injections, respectively. (**b**, **c**) Graphs showing the total distance traveled and time spend in corners (%) of the open field. (**b**) After 5 days of DT, CRE + DTR + mice exhibit significant reduced distance traveled compared to CTL mice. Time spent in the corners of the chamber did not differ between CRE + DTR + and CTL mice. (**c**) 30 days after the last DT injection no differences are observed between CRE + DTR + and CTL mice, indicating a recovery of locomotor activity (**c**). (**d**, **e**) Graphs showing the total arm entries and percentage of alternation in the Y-maze test. During OPC ablation CRE + DTR + mice exhibit significant less entries and alternation (%) in the maze compared to CTL (**d**), but these parameters are restored 31 days after the last DT injection, showing no more difference between the two groups (**e**). Dotted line depicts chance level performance. All data are presented as mean ± SEM (dots = single animal value in each group; Mann–Whitney test; *p ≤ 0.05).

To further characterize this mouse model and investigate the functional role of OPC unrelated to myelin production, we examined the behavioral consequences of acute OPC depletion, specifically on locomotion, anxiety and spatial working memory (Fig. 4a).

The open field test was used to assess spontaneous locomotor/exploratory activity and anxiety-like behaviors^49^, based on the observation that mice show distinct aversions to large, brightly lit open and unknown environments^50^. Mice were tested immediately after 5 days DT injections (Fig. 4b). The total distance traveled during the 8 min session in the open field was significantly reduced in CRE + DTR + mice compared to the CTL group (CTL: 2190 ± 148 vs. CRE + DTR + : 1720 ± 182 cm, *p* = 0.046; Fig. 4b), as well as the velocity (not shown), indicating decreased locomotor activity. Although the frequency of crossing center area boundaries was significantly decreased in CRE + DTR + mice (not shown), there was no difference between the two groups in the proportion of time spent in the center, along the border and in the corners of the chamber, indicating no signs of anxiety (Fig. 4b). Mice were then tested one month after the last DT injection in the same paradigm (Fig. 4c). At this time point, the two groups did not differ in the total distance traveled, showing locomotor activity recovery of the CRE + DTR + mice (Fig. 4c).

Spontaneous alternation in the Y-maze is an optimal test to assess spatial working memory^51^. The test is based on the natural tendency of mice to prefer exploring a novel arm over a familiar one, which leads them to alternate the choice of the arm visited across repeated trials. Mice were tested immediately after 7 days DT injection. CRE + DTR + mice exhibited reduced total arm entries (CTL: 29 ± 2 vs. CRE + DTR + : 22 ± 2, *p* = 0.03; Fig. 4d), consistent with the reduced activity observed in the open field. Independently of the exploratory activity, the percentage of alternation was significantly lower in OPC-deleted mice compared to the control (CTL: 66 ± 2 vs. CRE + DTR + : 58 ± 3, *p* = 0.02; Fig. 4d), indicating impaired spatial working memory. When tested again one month after the end of DT administration, mice of the two groups showed no more significant differences in their scores for the total arm entries and the percentage of alteration. However, the tendency remained for total arm entries (Fig. 4e).

These results suggest that OPC depletion does not influence anxiety but results in reduced locomotor activity (distance and speed) and altered spatial working memory. Interestingly, these effects are reversible with OPC density recovery.

### Acute OPC depletion results in decreased excitatory input to layer 5 pyramidal neurons

**Figure 5:**
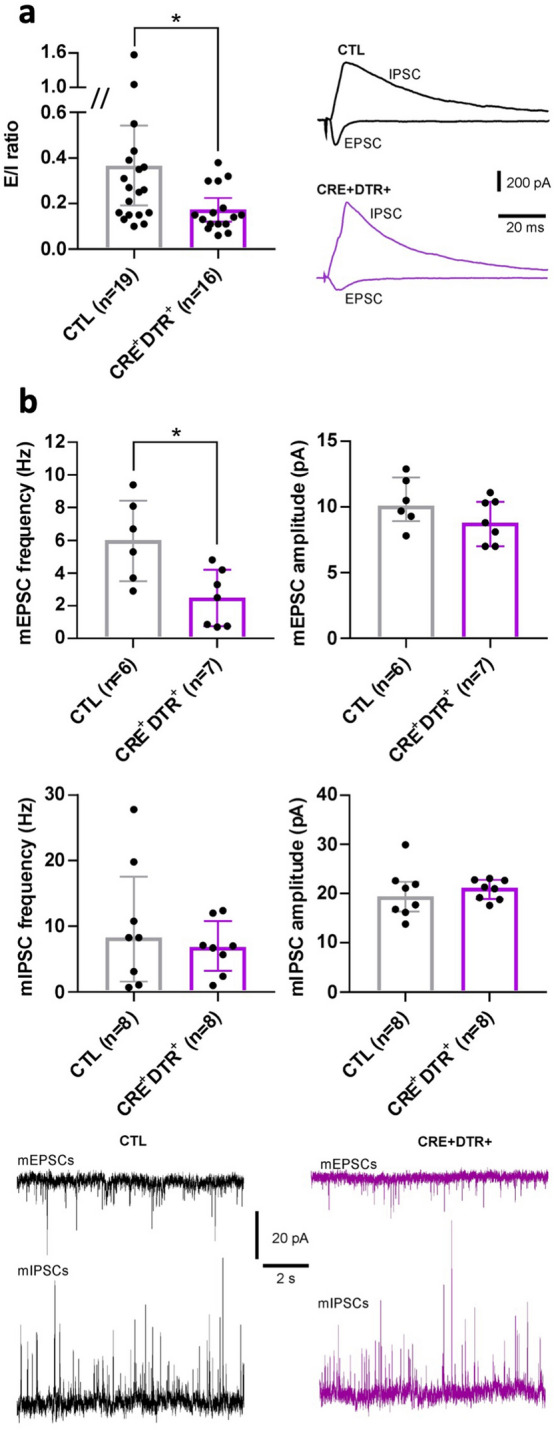
OPC depletion results in decreased excitatory glutamatergic input at the level of L5 CPNs. (**a**) The E/I ratio recorded in L5 CPNs is significantly decreased in CRE + DTR + mice compared to CTL (Mann–Whitney test; *p ≤ 0.05). The traces show sample IPSCs and EPSCs recorded from a CTL (black) and a CRE + DTR + (purple) CPN at a holding potential of 0 and − 60 mV, respectively. (**b**) The frequency of mEPSCs is significantly lower in CRE + DTR + mice compared to CTL (Mann–Whitney test; *p < 0.05). Traces depict samples of miniature activity recorded from a CTL (black) and a CRE + DTR + (purple) CPN. Data are presented as median ± interquartile range, dots show single neuron values; n refers to the number of recorded CPNs, which were obtained from 7 CTL and 6 CRE + DTR + mice.

The above-described behavioral deficits, particularly the reduced locomotor activity, could be linked to neuronal dysfunction at the level of the sensorimotor CX. Therefore, to test whether OPC depletion affects the cortical synaptic network, we recorded excitatory (glutamatergic) and inhibitory (GABAergic) synaptic currents converging on L5 CPNs, triggered by electrical stimulation of afferent fibers arising from L2-3 neurons and local interneurons, respectively, after 5 days of DT injections in slices from CTL and CRE + DTR + mice. We calculated the excitation-inhibition (E/I) ratio in each recorded CPN as the amplitude of AMPA receptor-mediated glutamatergic excitatory postsynaptic currents (EPSCs) to GABA_A_ receptor-mediated inhibitory postsynaptic currents (IPSCs). EPSCs (but not IPSCs) were blocked by 10 µM CNQX, and IPSCs (but not EPSCs) by 50 µM picrotoxin, confirming that they were actually mediated by AMPA and GABA_A_ receptors, respectively, and that IPSCs did not originate from feed-forward circuits (not shown). Interestingly, the E/I ratio was significantly decreased in CPNs from CRE + DTR + mice compared to CTL (*p* = 0.0144; Fig. 5a). This result can be attributable to decreased glutamate release, increased GABA release, or both, as well as to postsynaptic changes at the level of AMPA and/or GABA_A_ receptors. In order to address this issue, we also recorded spontaneous synaptic activity from the same CPNs used for calculating E/I ratio. However, we did not observe any significant change in either spontaneous EPSCs (sEPSCs) or IPSCs (sIPSCs) amplitude and frequency between CPNs from control and CRE + DTR + mice (Supplementary Fig. S6). On the other hand, the frequency of miniature EPSCs (mEPSCs) was significantly reduced in CRE + DTR + mice compared to control (*p* = 0.0221; Fig. 5b) while their amplitude was not affected, suggesting a decrease of glutamate release probability. Moreover, the frequency and amplitude of miniature IPSCs (mIPSCs) were similar between the two experimental groups (Fig. 5b).

These data show that the E/I ratio in the sensorimotor CX is drastically reduced after OPC depletion, which could underlie the behavioral phenotype of CRE + DTR + mice.

## Discussion

Apart from the localized germinative niches, OPC are the main proliferative cells of the healthy adult CNS. They are widely distributed in all the parenchyma, both in the white and the grey matter. Besides their well-documented role in myelin remodeling and regeneration, increasing evidence of their close interaction with neurons and their contribution to the glial syncytium suggests other functions of OPC in health and disease. In order to get information on OPC functions, we generated a new mouse line enabling the specific ablation OPC in a timely controlled manner.

### Generation of the PDGFRaDTR mice, a new tool to study OPC function in health and disease

Specific targeting is critical when trying to decipher a cell population's function. Several laboratories developed methodological approaches and models to delete OPC^42–44,52^, but none is specific and thus may lead to incorrect interpretation. This year, Merson’s lab reported a novel approach combining genetic and pharmacologic treatment to specifically and durably delete both parenchymal and subventricular-derived OPC^45^. However, this new mouse model has not been functionally characterized yet. Our model ensures specificity by double selection since PDGFRa and Olig2 promoters drive DTR expression. After crossing our PDGFRa-VenusStopFlox-DTR mice with Olig2Cre mice, some blood vessels are still Venus + , indicating that control under PDGFRa promoter only is insufficient to reach the required specificity. The controlled expression under the Olig2 promoter permits to target the oligodendrocyte lineage in the brain exclusively, and the control under the PDGFRa promoter restricts expression to immature progenitors, thus excluding OLG.

While characterizing our new mouse line, we identified two types of mosaicism at the cellular level. First, we observed rare single cells expressing Venus in CRE + DTR + mice not injected with DT, suggesting that Cre recombination is not 100% efficient. Could these rare DT-insensitive cells mediate OPC repopulation and explain that long-term DT injection failed to maintain depletion? The increased size and number of Venus + cell patches observed in CRE + DTR + mice following DT injections sustains such hypothesis. Upon DT injection, the reactivity of a single Venus + OPC may give rise to a clone of resistant OPC that may, in turn, repopulate the area. In parallel, in the absence of CRE expression, i.e. in CRE-DTR + mice where all OPC should be Venus + , we observed areas where PDGFRa + cells were Venus-, which suggests that not all OPC do express the PDGFRaDTR allele. Given that we worked with heterozygous PDGFRaDTR mice, PDGFRa KO being embryonic lethal^53–55^, we suspect that during development, randomly, some OPC may express only the PDGFRa wild-type allele and give rise to entire clones of Venus- OPC as they colonize the brain and proliferate. This process is called monoallelic gene expression. In the mouse, 6 to 10% of genes are randomly targeted with epigenetic modifications that regulate chromatin accessibility, result in transcriptional modification^56^, and give rise to cellular mosaicism^57^ in a clonal cell lineage within the same tissue^58^. Thus, these Venus- OPC are also DT insensitive and could participate in OPC repopulation: gradually, during DT treatment, resistant Venus- OPC replace lost OPC, and finally, all OPC become DT insensitive.

### Specific but transient OPC ablation in the adult mouse brain

We show here that 2 or 7 days of daily DT injection induces a strong and significant reduction in OPC density in the CC and CX without affecting mature OLG. Despite significant loss of OPC, we did not detect any overt inflammation in the brain since microglia were not activated after DT injections and astrocytes did not change morphology or show increased GFAP expression. Such absence of glial reactivity has also been reported in other models that induce OPC death^42,44,59^, although a modest and transient astrocytic and microglial activation was observed when associating genetic and pharmacological approaches to kill OPC and prevent cell proliferation^45^. The reduced microglia activation was not associated with an increase in proinflammatory cytokine expression level^45^. OPC depletion was more robust in the CX compared to CC, an effect already observed^42^ that could be linked either to intrinsic OPC heterogeneity or environmental factors. It is not clear whether OPC in the CX are more susceptible to DT-induced cell death and/or whether spared OPC in the CX are less reactive to compensate for their neighbor loss. Indeed, OPC in the CX divide more slowly than in the CC^60,61^, a property that may slow down the repopulation in the CX. In their recent paper, Xing et al. successfully combined genetic and pharmacological approaches to kill OPC and suppress cell proliferation to prevent OPC repopulation^45^, but the infusion of the antiproliferative agent AraC utilized in this study induced weight loss and non-specific clinical signs that may also impact behavioral analyses.

In our model, the strong and significant reduction in OPC density obtained after 2 or 7 days of daily DT injection is transient. Indeed, 7 days after the last injection, OPC density returned almost to control levels, suggesting that the surviving OPC keep a high proliferation and compensation potential. We tried sustained DT injections over long periods with different protocols but failed to maintain the ablation level. In physiological conditions, OPC density homeostasis is finely regulated through a balance of active growth and self-repulsion^2^. A recent study showed that PDGFRa inactivation led to OPC elimination due to their synchronous differentiation into OLG, but the OPC population was robustly reconstituted by active expansion of Nestin + pericytes, Nestin + progenitors from meninges, and by few OPC that escaped from inactivation^62^. Using BrdU tracing before DT injections to label SVZ-derived progenitors, we checked if the OPC generated in the SVZ could be responsible for OPC replenishment in the CC (not shown). We detected no substantial SVZ cell exit and migration in the CC. Thus, this impressive recovery is most likely due to spared OPC that escaped cell death and repopulated the CC and the CX. The recovery of OPC density is common to all ablation paradigms tested (for review, see^63^), highlighting the self-renewal stem cell-like properties of OPC. To achieve long-term OPC ablation, Xing et al. infused AraC in the cisterna magna to block cell proliferation, but doing so, they also affected neuroblast proliferation. Contrary to our results, they observed PDGFRa + Nestin + OPCs in the CC during recovery and concluded that OPC repopulation is driven by SVZ-derived progenitors^45^.

### OPC ablation leads to behavioral impairment and disruption of the excitatory to inhibitory balance in the sensorimotor cortex

Mice with OPC depletion showed reduced locomotor activity, as revealed in the open field and the Y-maze. The decreased velocity and time spent in movement may indicate the fatigability of these mice. This reduced activity may also reflect a decreased attraction for exploration. Indeed, previous studies attempting to ablate OPC described increased depressive-like behaviors^42^. Our study did not detect signs of anxiety in OPC-depleted mice compared to CTL mice as assessed in the open field. In contrast, OPC ablation resulted in reduced alternation in the Y-maze, indicative of altered spatial working memory. Because all groups of mice were injected with DT we can rule out a non-specific toxic effect of DT to be responsible for differences observed between CTL and OPC-depleted mice. Besides, because of the highly specific expression of DTR in the CNS and in OPC, this phenotype is unlikely to derive from peripheral or muscular alterations. However, we cannot exclude that damage to OPC in the spinal cord may contribute to the phenotype. Also, despite low inflammatory response, we cannot ignore that OPC ablation may disturb tissue homeostasis and indirectly impact E/I balance and behavior. Further experiments perturbing OPC physiology without killing them would be required to rule out this possibility.

Since previous work provided evidence that OPC could regulate information processing at neuronal synapses^36,39,42^, we examined the impact of acute OPC ablation on excitatory (AMPA receptor-mediated) and inhibitory (GABA_A_ receptor-mediated) synaptic transmission converging on L5 CPNs, using whole-cell patch-clamp recordings on acute brain slices. Our data suggest that the excitatory input to L5 CPNs is reduced in the sensorimotor CX of CRE + DTR + mice, as demonstrated by the significant decrease of both E/I ratio and mESPC frequency. Since neither mEPSC amplitude nor mIPSC parameters are changed, such reduced excitatory synaptic activity at the level of L5 CPNs seems due mainly to a decreased probability of glutamate release from presynaptic fibers targeting these neurons. In this context, it is interesting to note that the partial ablation of OPC in stress-susceptible mice has been shown to diminish glutamatergic signaling in prefrontal CX^42^. This change could profoundly alter the way synaptic signaling is processed in this structure and the circuitry involving L5 CPNs, which might contribute to the altered phenotype of CRE + DTR + mice. Moreover, recent data has highlighted a new function of OPC that can participate in synapse engulfment, which may regulate synaptic connectivity in the brain^36^. Finally, a recent paper showed that GABA released from NG2 glia activation enhanced inhibitory transmission onto proximal hippocampal interneurons^41^. However, such a mechanism does not concern the CX in our CRE + DTR + mouse model, since GABA_A_ receptor-mediated synaptic input is unchanged at the level of L5 CPNs.

Our results suggest that acute OPC ablation impacts behavior and cognitive functions, possibly via a shift in the E/I balance in the cerebral CX. Because of the short duration of ablation correlated with behavioral deficits and the restoration of behavior with recovery of OPC density, it is unlikely that this effect is due to myelination dysfunction. These data thus reinforce the hypothesis of a physiological role of OPC in modulating neuronal activity and brain function.

## Material and methods

All experimental and surgical protocols were performed following the guidelines established by the French Ministry of Agriculture (Animal Rights Division). The architecture and functioning rules of our animal house, as well as our experimental procedures have been approved by the “Direction Départementale des Services Vétérinaires” and the ethic committee (ID numbers F1305521 and 2016071112151400 for animal house and research project respectively).

### Construction of targeting vector and generation of the PDGFRa-VenusStopFlox-DTR mice

To specifically delete OPC, a targeting vector (17.6 kb) containing the human DTR was introduced in the exon 2 of the PDGFRa gene on the chromosome 5, by homologue recombination in embryonic stem (ES) cells. Two plasmids from Moqrich A. laboratory were used to construct the targeting vector: (1) pGEMT plasmid carrying DTR and the neomycin (NEO) resistance cassette, to further select ES, flanked by two FRT site; (2) EGFP-N1 plasmid carrying venus flanked by two loxp site and polyA sequences. Using cloning strategy DTR and NEO sequence were cloned in the EGFP-N1 plasmid next to venus and 3polyA sequences. To ensure homologous recombination in ES cells, a long 5’ arm (> 5.5 Kb upstream the ATG in exon 2 of PDGFRa gene) and 3’ arm (3.1 Kb downstream ATG) were cloned in the EGFP-N1 plasmid (Supplementary Fig. S1a).

After AatII (New England Biolabs) digestion, the linearized plasmid was introduced by electroporation into embryonic stem cells (ES CK35 from 129/SvPas background) by TAAM-SEAT (Service des Animaux Transgéniques-Villejuif). NEO resistant ES cell clones were checked for homologous recombination by a southern blot using a ^32^P labeled 5’ external probe (598 pb) used after ApaI (New England Biolabs) DNA digestion, and 3’ external probe (752 pb) used after BamH1 (New England Biolabs) DNA digestion (Supplementary Table S2). Only one targeted ES cell clone was obtained with complete homologous recombination (Supplementary Fig. S1b). This positive ES cell clone was injected into C57BL/6 blastocysts and gave rise to germline chimeras.

PDGFRa-Venusstopflox-DTR mice were crossed with Actin Flipase mice to remove NEO cassette (Supplementary Fig. S1a). Once NEO cassette was eliminated, mice negative for flipase gene were selected. Finally, as PDGFRa is expressed in conjunctive tissue and in periphery, PDGFRaDTR mice were crossed with Olig2CRE mice^48^ to ensure specificity in the CNS and in the oligodendroglial lineage. Thus, to generate experimental cohorts, heterozygous PDGFRa-VenusStopFlox-DTR females were crossed with Olig2Cre males. In the Olig2CRE:PDGFRaDTR mice, CRE recombinase triggers the excision of Venus together with the stop sequence, allowing DTR expression under the control of PDGFRa promoter. We thus obtained with these breedings 4 groups of mice in our experimental cohorts (Supplementary Fig. S1a): CRE-DTR-, CRE-DTR + , CRE + DTR- (these 3 groups are controls and will be referred to as “CTL”) and CRE + DTR + (mice of interest, in which OPC express DTR).

To preserve and maintain our newly generated mouse line, heterozygous PDGFRa-VenusStopFlox-DTR mice were crossed with C57BL/6.

### Genotyping of transgenic mice

First, genomic DNA of the founders was isolated form tail biopsies using tail lysis buffer containing 100 mM Tris–HCL (pH8.0), 200 mM NaCl, 5 mM EDTA (pH8), 0.2% SDS, and proteinase K 100 µg/ml. After isopropanol precipitation, genomic DNA was checked with long PCR with primers overlapping PDGFRa5’ region and venus, and also with primers overlapping NEO and PDGFRa3’ region, using Long Amp polymerase (New England Biol). Once the colony well established, then for routine analysis genomic DNA was isolated without precipitation from tail biopsies using tail lysis buffer containing 10 mM Tris–HCl (pH 8.5), 50 mM KCl, 1.5 mM MgCl_2_, 0.01% gelatin, 0.45% NP40, 0.45% Tween 20 and proteinase K 100 µg/ml. After proteinase K inactivation and centrifugation, 2 µl of crude lysate was directly used in PCR reaction. Platinum Green Hot Start PCR Master mix was used (Invitrogen). The primer sequences and PCR protocols are detailed in Supplementary Table S3. PCR reaction was performed on thermal cycler (2720; Life Technologies). DNA fragments were visualized after electrophoresis of PCR products in 2% agarose gel, or 0.8% for long PCR products, stained with ethidium bromide under UV illumination.

### Diphtheria toxin injections

DT toxin was purchased from Merck. Upon arrival DT toxin was dissolved in sterile PBS (stock solution: 100 µg/ml) and aliquots were conserved at -80 °C. Dilutions for injection protocols were aliquoted and conserved at -20 °C to avoid thaw and freeze cycles. DT was injected either IP or SC.

Mice between 7 and 11-week-old were injected with 10 ng/g, 20 ng/g or 40 ng/g once a day during 2 or 7 days. Mice were sacrificed 1 day, 7 days or 1 month after the last DT injection. In some experiments, SC injections of DT were maintained for longer periods (Supplementary Fig. S3). In all experiments, mice from all groups (CTL and CRE + DTR + mice) were injected with DT.

### Behavioral tests

#### Open field test

The open field chamber measured 40 × 40 cm and was made of white high-density non-porous plastic. The chamber was wiped with 30% ethanol before testing each mouse to remove any odor cues and light was controlled. Mice were video-tracked using EthoVision software XT 16 (Noldus Inc.). Calibration was performed according to manufacturer’s instructions. Mice were familiarized with the room at least 45 min before the test. They were placed in the center of the chamber and were allowed to freely move and explore the arena for 8 min. To analyze mice behavior during this test, the chamber was divided in zones: center, border and corners. Parameters measured were: total distance traveled, locomotion speed, time and frequency of crossing in the three different zones.

#### Y-maze spontaneous alternation test

Y-maze was made of white high-density non-porous plastic (Noldus). All maze arms are 35 long × 6 wide × 15 high cm. Three equal 120° angles separate each arm. Mice were video-tracked using EthoVision software XT 16 (Noldus Inc.) and the alternation was manually scored from the videos. As for the open field test, mice were put in the room at least 45 min before, the apparatus was cleaned with 30% ethanol between each mouse and light was controlled. Mice were placed in the starting arm facing the center and were allowed to freely explore the maze for 8 min. Scoring alternations is based on triplets. An alternation is scored when all three arms are explored consecutively. The first two arm visits can be considered samples. For all the following visits, an entrance is rated alternation if the chosen arm has not been visited in the two previous choices. We used the percentage of alternation as an index of spatial working memory using the following formula: % of alternation = [number of alternation/(total number of entries -1)]*100.

### Ex vivo patch-clamp recordings

Procedures were similar to those described previously^64^. 6–7 weeks old CTL (n = 7) and CRE + DTR + mice (n = 6) were injected with DT (20 ng/g, SC) for 5 days. After sacrifice, acute coronal brain slices (250 µm-thick) were obtained using a vibratome (Leica S1000) in ice-cold solution containing (in mM): 110 choline, 2.5 KCl, 1.25 NaH_2_PO_4_, 7 MgCl_2_, 0.5 CaCl_2_, 25 NaHCO_3_, 7 glucose, pH 7.4. Slices were then kept at room temperature in oxygenated artificial cerebrospinal fluid (ACSF), whose composition was (in mM): 126 NaCl, 2.5 KCl, 1.2 MgCl_2_, 1.2 NaH_2_PO_4_, 2.4 CaCl_2_, 11 glucose, and 25 NaHCO_3_, pH 7.4, at 34–35 °C, flowing at ~ 2.5 ml/min. For electrophysiological recordings, slices were individually transferred in oxygenated ACSF at 32-33°C flowing at 2–3 ml/min. L5 CPNs of the primary motor and somatosensory CX were identified by infrared videomicroscopy and by their electrophysiological properties^65^, and recorded in whole-cell patch-clamp by borosilicate micropipettes (4–5 MΩ), filled with an internal solution containing (in mM): 126 mM CsCH_3_SO_3_, 10 mM HEPES, 1 mM EGTA, 2 mM QX-314 chloride, 0.1 mM CaCl_2_, 4 mM Mg-ATP, 0.3 mM Na-GTP, pH 7.3. This solution allows recording AMPA receptor-mediated excitatory postsynaptic currents (EPSCs) or GABA_A_ receptor-mediated inhibitory postsynaptic currents (IPSCs) by voltage-clamping the recorded neuron around -60 or 0 mV, respectively. A stimulating bipolar electrode was placed in the CX at the level of L4 to activate local fibers and evoke EPSCs and IPSCs, which were recorded in each CPN on consecutive sweeps triggered every 10 s. Input resistance was monitored in each sweep by sending a 5 mV pulse, and cells were discarded if this parameter changed by > 20%. The amplitude of 6 consecutive EPSCs and IPSCs was measured, and the E/I ratio was calculated as the average EPSC amplitude divided by the average IPSC amplitude for each recorded CPN. Spontaneous EPSCs and IPSCSs (sEPSCs and sIPSCs, respectively) were recorded for 1–2 min at a holding potential around − 60 and 0 mV, respectively. Miniature EPSCs and IPSCs (mEPSCs and mIPSCs, respectively) were recorded similarly, but in the presence of 1 µM tetrodotoxin applied for at least 5 min. In some CPNs, spontaneous and miniature EPSC and/or IPSCs were not detectable, hence the different number of samples reported in the graphs. For analyzing spontaneous and miniature activity, the detection threshold was set to twice the noise after trace filtering (Boxcar low-pass), and only cells exhibiting stable activity and baseline were taken into account. Electrophysiological data were acquired by an AxoPatch 200B amplifier coupled to a Digidata 1550B interface using pClamp 10.7 (Axon Instruments/Molecular Devices, USA), and analyzed offline using Clampfit 10.7 (Molecular Devices, USA) and MiniAnalysis 6.0 (Synaptosoft, USA) software. Statistical analysis was performed using Prism 7.05 (GraphPad Software, USA). CTL data were obtained from CRE-DTR- and CRE + DTR- mice; Mann-Whiney test revealed no significant difference between CRE-DTR- vs. CRE + DTR- values, thus they were pooled as CTL.

### Tissue preparation and immunohistochemistry

After intracardiac perfusion with 4% paraformaldehyde, brains were removed and postfixed for 2 h. After PBS wash, brains were cut on a vibratome (HM 650 V; Microm) on serial coronal 50 µm thick sections. We used the following primary antibodies: chicken anti-GFP (1/500; Aves Labs), rabbit anti-Olig2 (1/500; Chemicon), rat anti-PDGFRa (CD140a) (1/250; Millipore), rat anti-CD68 (1/200; Abcam), and rabbit anti-GFAP (1/500; Dako).

### Microscopy and quantification

Images were captured with a Zeiss apotome system (20 × objective). CC and CX were analyzed in two rostro-caudal locations: above the rostral SVZ (Bregma + 0.5 to + 1 mm) and at the fornix level (Bregma − 0.3 to − 0.8 mm). A minimum of 3 sections for each location were analyzed per mice. The area of interest was delineated and the cells were manually counted using Zen software (Zeiss) (for PDGFRa + cells) or using automated counting with ImageJ software (for Olig2 + cells).

### Statistical analyses

Non-parametric tests were performed using Prism 8.4.3 (GraphPad Software, USA). We used the Kruskal–Wallis test followed by Dunn’s multiple comparison to compare CRE + DTR + mice with the three CTL groups. We used the Mann–Whitney test to compare CRE + DTR + mice with pooled CTL mice. Chi-square test was used to analyze the number and category of Venus + patches over time.

### Supplementary Information

Supplementary Information.

## Data Availability

The data that support the current study are available from the corresponding author on reasonable request.

## References

[1] Rowitch DH, Kriegstein AR (2010). Developmental genetics of vertebrate glial-cell specification. Nature.

[2] Hughes EG, Kang SH, Fukaya M, Bergles DE (2013). Oligodendrocyte progenitors balance growth with self-repulsion to achieve homeostasis in the adult brain. Nat. Neurosci..

[3] Kirby BB (2006). In vivo time-lapse imaging shows dynamic oligodendrocyte progenitor behavior during zebrafish development. Nat. Neurosci..

[4] Dawson M (2003). NG2-expressing glial progenitor cells: An abundant and widespread population of cycling cells in the adult rat CNS. Mol. Cell. Neurosci..

[5] Psachoulia K, Jamen F, Young KM, Richardson WD (2009). Cell cycle dynamics of NG2 cells in the postnatal and ageing brain. Neuron Glia Biol..

[6] Wolswijk G, Noble M (1989). Identification of an adult-specific glial progenitor cell. Development.

[7] Tang DG, Tokumoto YM, Raff MC (2000). Long-term culture of purified postnatal oligodendrocyte precursor cells. Evidence for an intrinsic maturation program that plays out over months. J. Cell Biol..

[8] Gensert JM, Goldman JE (1997). Endogenous progenitors remyelinate demyelinated axons in the adult CNS. Neuron.

[9] Franklin RJM, Gilson JM, Blakemore WF (1997). Local recruitment of remyelinating cells in the repair of demyelination in the central nervous system. J. Neurosci. Res..

[10] Zawadzka M (2010). CNS-resident glial progenitor/stem cells produce schwann cells as well as oligodendrocytes during repair of CNS demyelination. Cell Stem Cell.

[11] Orthmann-Murphy J (2020). Remyelination alters the pattern of myelin in the cerebral cortex. eLife.

[12] Flygt J, Djupsjö A, Lenne F, Marklund N (2013). Myelin loss and oligodendrocyte pathology in white matter tracts following traumatic brain injury in the rat. Eur. J. Neurosci..

[13] Behrendt G (2013). Dynamic changes in myelin aberrations and oligodendrocyte generation in chronic amyloidosis in mice and men. Glia.

[14] Adams KL, Gallo V (2018). The diversity and disparity of the glial scar. Nat. Neurosci..

[15] Zhu X (2011). Age-dependent fate and lineage restriction of single NG2 cells. Development.

[16] Boda E (2015). Early phenotypic asymmetry of sister oligodendrocyte progenitor cells after mitosis and its modulation by aging and extrinsic factors. Glia.

[17] Gibson EM (2014). Neuronal activity promotes oligodendrogenesis and adaptive myelination in the mammalian brain. Science.

[18] Simon C, Götz M, Dimou L (2011). Progenitors in the adult cerebral cortex: Cell cycle properties and regulation by physiological stimuli and injury. Glia.

[19] McKenzie IA (2014). Motor skill learning requires active central myelination. Science.

[20] Xiao L (2016). Rapid production of new oligodendrocytes is required in the earliest stages of motor-skill learning. Nat. Neurosci..

[21] Pan S, Mayoral SR, Choi HS, Chan JR, Kheirbek MA (2020). Preservation of a remote fear memory requires new myelin formation. Nat. Neurosci..

[22] Steadman PE (2020). Disruption of oligodendrogenesis impairs memory consolidation in adult mice. Neuron.

[23] Yeung MSY (2014). Dynamics of oligodendrocyte generation and myelination in the human brain. Cell.

[24] Tripathi RB (2017). Remarkable stability of myelinating oligodendrocytes in mice. Cell Rep..

[25] Bacmeister CM (2020). Motor learning promotes remyelination via new and surviving oligodendrocytes. Nat. Neurosci..

[26] Bergles DE, Roberts JD, Somogyi P, Jahr CE (2000). Glutamatergic synapses on oligodendrocyte precursor cells in the hippocampus. Nature.

[27] Lin S, Bergles DE (2004). Synaptic signaling between GABAergic interneurons and oligodendrocyte precursor cells in the hippocampus. Nat. Neurosci..

[28] Mangin J-M, Kunze A, Chittajallu R, Gallo V (2008). Satellite NG2 progenitor cells share common glutamatergic inputs with associated interneurons in the mouse dentate gyrus. J. Neurosci..

[29] Müller J (2009). The principal neurons of the medial nucleus of the trapezoid body and NG2(+) glial cells receive coordinated excitatory synaptic input. J. Gen. Physiol..

[30] Lin S-C (2005). Climbing fiber innervation of NG2-expressing glia in the mammalian cerebellum. Neuron.

[31] Kukley M, Capetillo-Zarate E, Dietrich D (2007). Vesicular glutamate release from axons in white matter. Nat. Neurosci..

[32] Etxeberria A, Mangin J-M, Aguirre A, Gallo V (2010). Adult-born SVZ progenitors receive transient synapses during remyelination in corpus callosum. Nat. Neurosci..

[33] Bergles DE, Jabs R, Steinhäuser C (2010). Neuron-glia synapses in the brain. Brain Res. Rev..

[34] Moura DMS, Brennan EJ, Brock R, Cocas LA (2021). Neuron to oligodendrocyte precursor cell synapses: protagonists in oligodendrocyte development and myelination, and targets for therapeutics. Front. Neurosci..

[35] Xiao Y, Petrucco L, Hoodless LJ, Portugues R, Czopka T (2022). Oligodendrocyte precursor cells sculpt the visual system by regulating axonal remodeling. Nat. Neurosci..

[36] Auguste YSS (2022). Oligodendrocyte precursor cells engulf synapses during circuit remodeling in mice. Nat. Neurosci..

[37] Buchanan J (2022). Oligodendrocyte precursor cells ingest axons in the mouse neocortex. Proc. Natl. Acad. Sci. U. S. A..

[38] Lepiemme F (2022). Oligodendrocyte precursors guide interneuron migration by unidirectional contact repulsion. Science.

[39] Sakry D (2014). Oligodendrocyte precursor cells modulate the neuronal network by activity-dependent ectodomain cleavage of glial NG2. PLoS Biol..

[40] Káradóttir R, Hamilton NB, Bakiri Y, Attwell D (2008). Spiking and nonspiking classes of oligodendrocyte precursor glia in CNS white matter. Nat. Neurosci..

[41] Zhang X (2021). NG2 glia-derived GABA release tunes inhibitory synapses and contributes to stress-induced anxiety. Nat. Commun..

[42] Birey F (2015). Genetic and stress-induced loss of NG2 Glia triggers emergence of depressive-like behaviors through reduced secretion of FGF2. Neuron.

[43] Montagne A (2018). Pericyte degeneration causes white matter dysfunction in the mouse central nervous system. Nat. Med..

[44] Schneider S (2016). Decrease in newly generated oligodendrocytes leads to motor dysfunctions and changed myelin structures that can be rescued by transplanted cells: Reduced oligodendrogenesis in the adult brain. Glia.

[45] Xing YL (2023). High-efficiency pharmacogenetic ablation of oligodendrocyte progenitor cells in the adult mouse CNS. Cell Rep. Methods.

[46] Marques S (2018). Transcriptional convergence of oligodendrocyte lineage progenitors during development. Dev. Cell.

[47] Orr-Urtreger A, Lonai P (1992). Platelet-derived growth factor-A and its receptor are expressed in separate, but adjacent cell layers of the mouse embryo. Development.

[48] Dessaud E (2007). Interpretation of the sonic hedgehog morphogen gradient by a temporal adaptation mechanism. Nature.

[49] Seibenhener ML, Wooten MC (2015). Use of the open field maze to measure locomotor and anxiety-like behavior in mice. J. Vis. Exp..

[50] Choleris E, Thomas AW, Kavaliers M, Prato FS (2001). A detailed ethological analysis of the mouse open field test: Effects of diazepam, chlordiazepoxide and an extremely low frequency pulsed magnetic field. Neurosci. Biobehav. Rev..

[51] Kraeuter A-K, Guest PC, Sarnyai Z (2019). The Y-maze for assessment of spatial working and reference memory in mice. Methods Mol. Biol..

[52] Chari DM, Crang AJ, Blakemore WF (2003). Decline in rate of colonization of oligodendrocyte progenitor cell (OPC)-depleted tissue by adult OPCs with age. J. Neuropathol. Exp. Neurol..

[53] Soriano P (1997). The PDGF alpha receptor is required for neural crest cell development and for normal patterning of the somites. Development.

[54] Hoch RV, Soriano P (2003). Roles of PDGF in animal development. Development.

[55] Tallquist MD, Soriano P (2003). Cell autonomous requirement for PDGFRalpha in populations of cranial and cardiac neural crest cells. Development.

[56] Massah S, Beischlag TV, Prefontaine GG (2015). Epigenetic events regulating monoallelic gene expression. Crit. Rev. Biochem. Mol. Biol..

[57] Branciamore S (2018). Frequent monoallelic or skewed expression for developmental genes in CNS-derived cells and evidence for balancing selection. Proc. Natl. Acad. Sci. U. S. A..

[58] Savova V (2016). Genes with monoallelic expression contribute disproportionately to genetic diversity in humans. Nat. Genet..

[59] Robins SC (2013). Evidence for NG2-glia derived, adult-born functional neurons in the hypothalamus. PLoS One.

[60] Rivers LE (2008). PDGFRA/NG2 glia generate myelinating oligodendrocytes and piriform projection neurons in adult mice. Nat. Neurosci..

[61] Young KM (2013). Oligodendrocyte dynamics in the healthy adult CNS: Evidence for myelin remodeling. Neuron.

[62] Đặng TC (2019). Powerful homeostatic control of oligodendroglial lineage by PDGFRα in adult brain. Cell Rep..

[63] Jäkel S, Dimou L (2017). Glial cells and their function in the adult brain: A journey through the history of their ablation. Front. Cell Neurosci..

[64] Chabbert D (2019). Postnatal Tshz3 deletion drives altered corticostriatal function and autism spectrum disorder-like behavior. Biol. Psychiatry.

[65] Hattox AM, Nelson SB (2007). Layer V neurons in mouse cortex projecting to different targets have distinct physiological properties. J. Neurophysiol..

