# Supplementary material for: Characterization of a new mouse line triggering transient oligodendrocyte progenitor depletion
Source: Sci Rep. 2023 Dec 11;13:21959. doi: 10.1038/s41598-023-48926-4 (PMC10713661; doi:10.1038/s41598-023-48926-4)
Supplement: Supplementary file 1 — Supplementary Information. [file 41598_2023_48926_MOESM1_ESM.pdf]

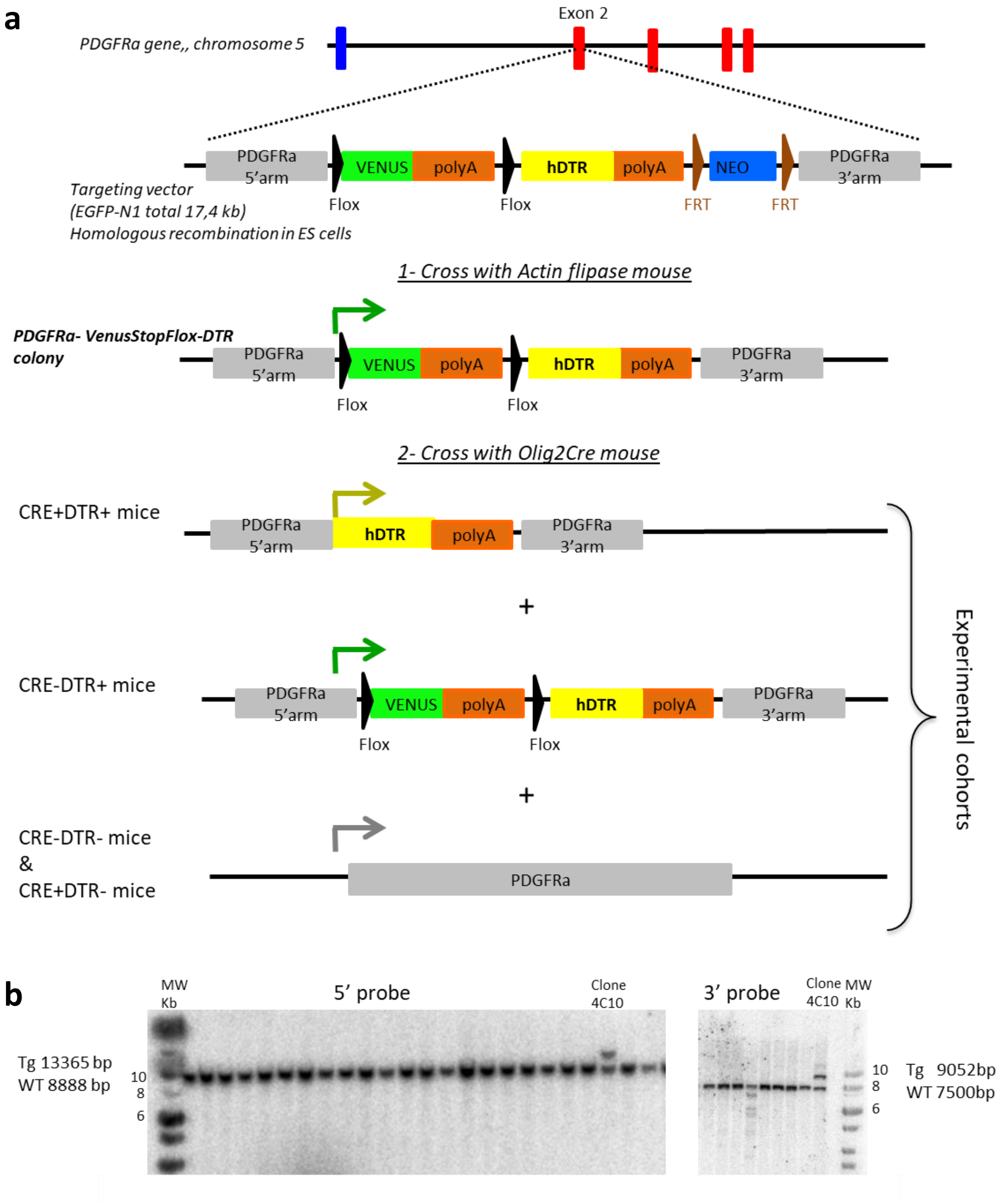

Supplementary Figure S1. Identification of embryonic stem cell clones with effective homologous recombination and generation of the PDGFRa-DTR mouse line. (**a**) Targeting vector inserted in exon 2 of PDGFRa gene by homologous recombination in mouse ES cells. After germ line transmission by the chimera mouse to its progeny, these mice were crossed with actin-Flipase mice to eliminate the Neo selection cassette. Finally, cohorts of mice for experimental use were obtained by crossing females from the PDGFRa-VenusStopFlox-DTR colony with Olig2Cre males in order to suppress the Venus-Stop cassette and allow DTR expression in Olig2+ PDGFRa+ cells (i.e. OPC). Four genotypes were obtained from these breedings: CRE+DTR+ mice (mice of interest, expressing DTR in OPC), CRE-DTR+ mice (expressing Venus in OPC), CRE+DTR- and CRE-DTR- mice (with wild type PDGFRa gene). (**b**) Southern blot from embryonic stem cell clones selected after electroporation of the construct of interest (PDGFRa5'arm-loxP-Venus-pA-loxP-hDTR-pA-FRT-Neo-FRT-PDGFRa3'arm). Only 1 clone (#4C10) showed the expected additional band with both the 5' probe (13365 bp) and with the 3' probe (9052 bp).

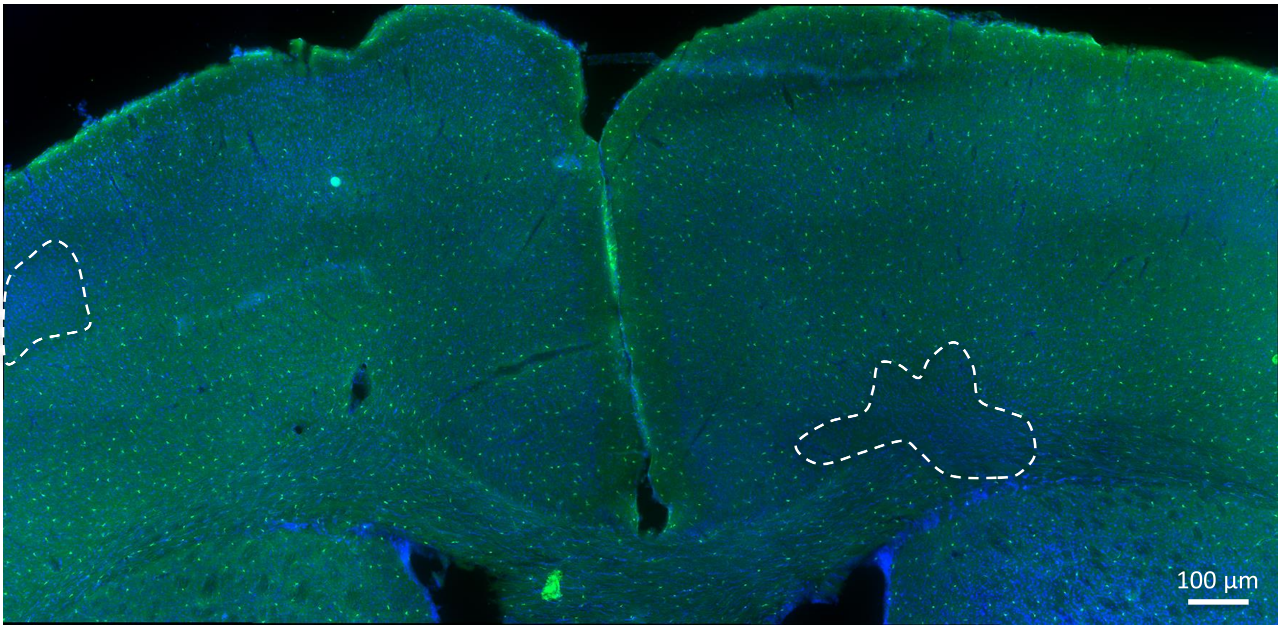

Supplementary Figure S2. Illustration of patches devoid of Venus+ cells in a CRE-DTR+ mouse. The patches are delimited by dotted lines.

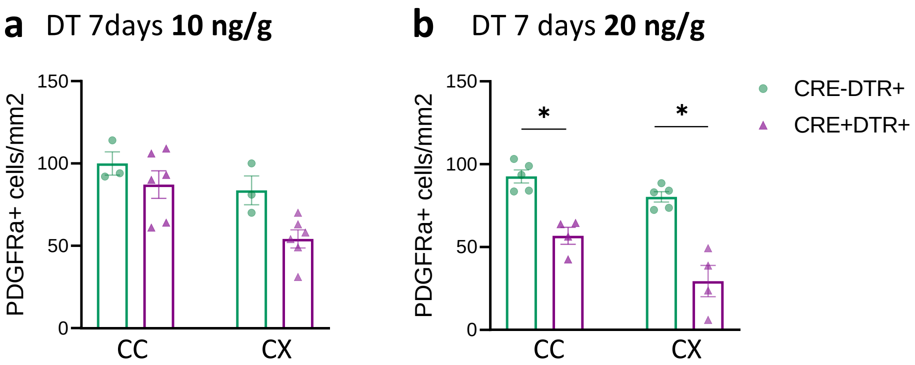

Supplementary Figure S3. Determination of the optimal dose of DT injection to obtain efficient OPC depletion. (**a**) One-week daily IP injection at 10 ng/g triggers non-significant reduction in OPC density in the CC and mild reduction in the CX. (**b**) One-week daily SC injection at 20 ng/g efficiently reduces OPC density in the CC and the CX. All data are presented as mean ± SEM (dots = single animal value in each group; Mann-Whitney test; *$p \leq 0.05$)

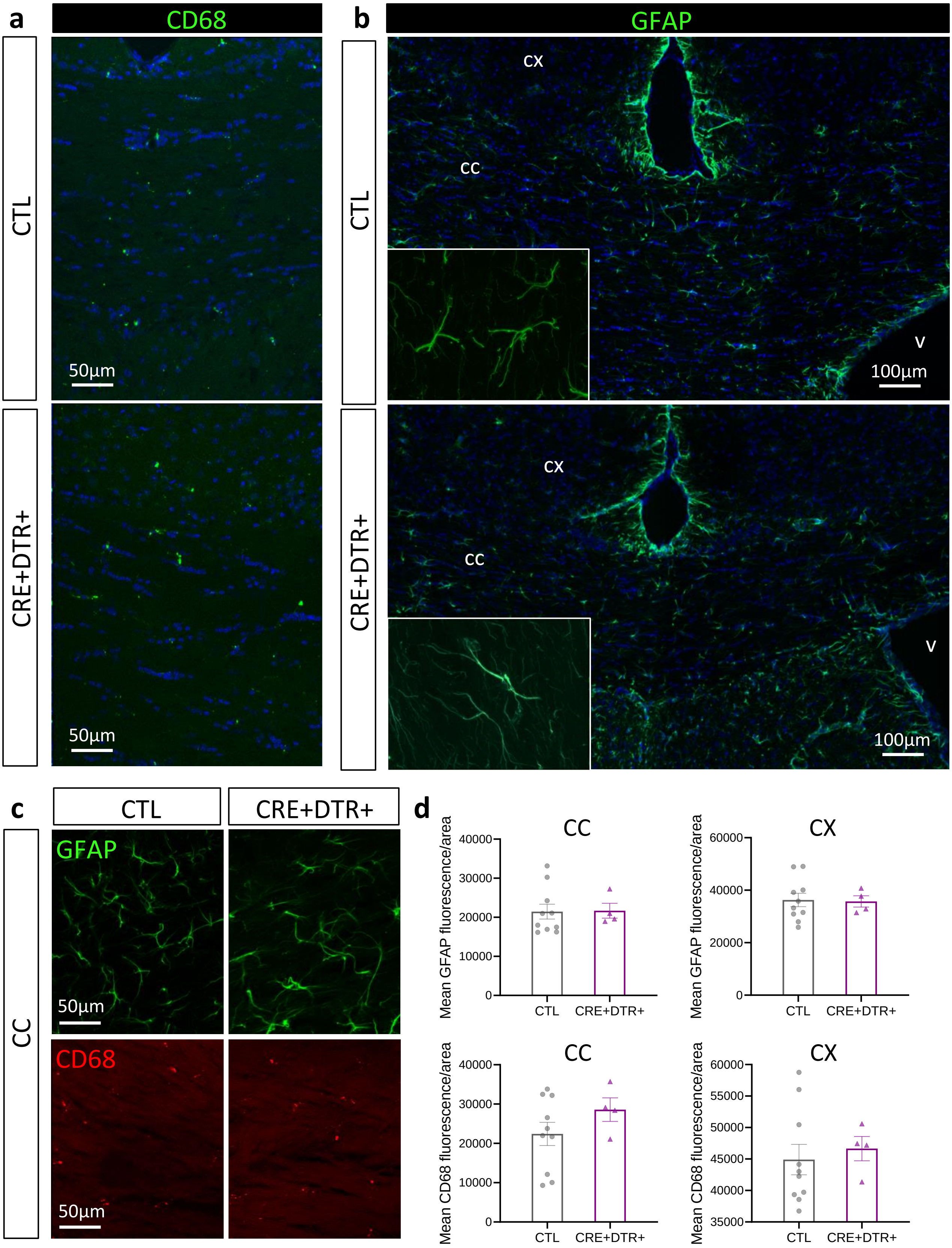

Supplementary Figure S4. DT-induced deletion of OPC does not trigger overt inflammation. CD68 (**a**) and GFAP (**b**) immunolabeling show no obvious microglial or astrocytic reactivity following one-week daily DT injections, nor after 2 days of DT injections (**c**, **d**) (dots = single animal value in each group; Mann-Whitney test; $p > 0.05$).

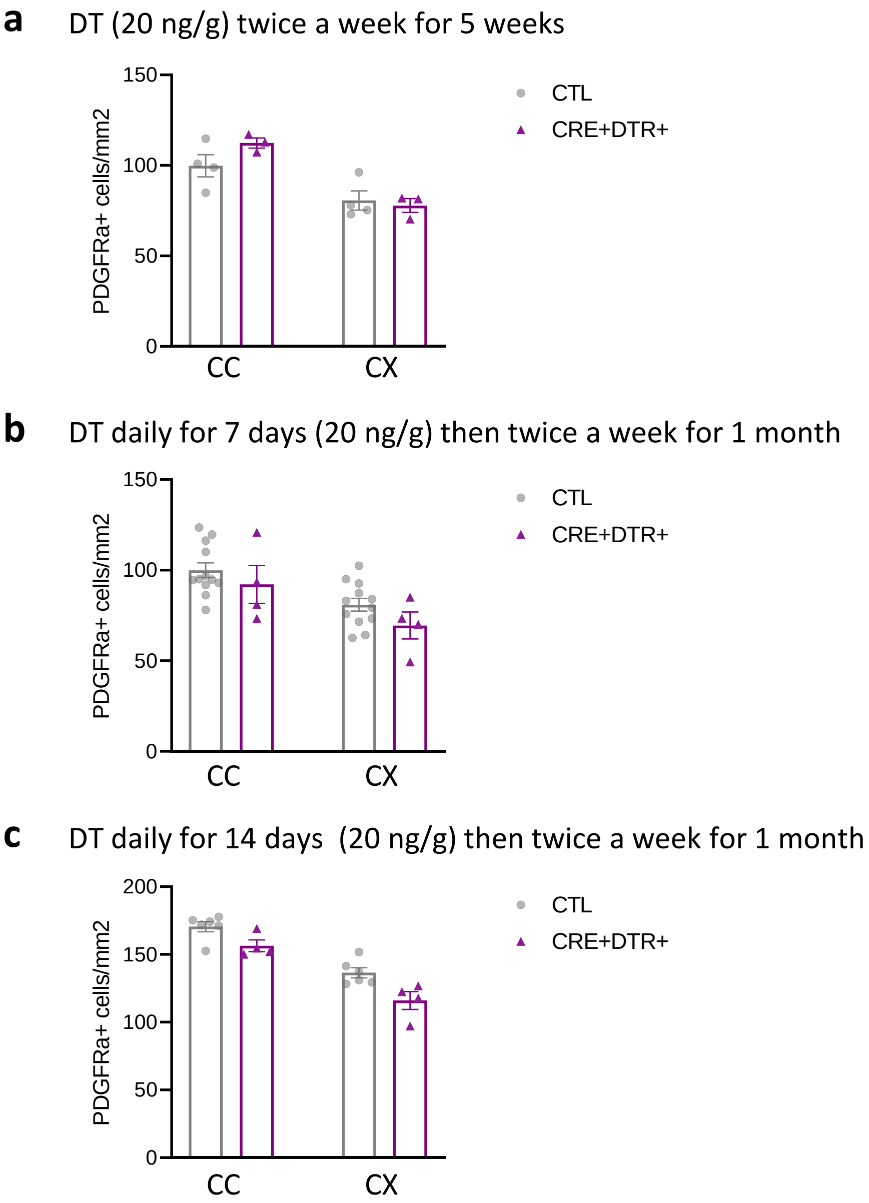

Supplementary Figure S5. Chronic DT injections are not sufficient to maintain OPC deletion on the long term. (**a**) OPC density is not reduced after 5 weeks DT treatment with 2 injections per week (20 ng/g). (**b**) First week with daily DT injection (20 ng/g) followed by 4 weeks with 2 injections per week does not maintain the initial deletion of OPC. (**c**) 2 weeks with daily DT injection (20 ng/g) followed by 4 weeks with 2 injections per week also fails to significantly reduce OPC density in CC and CX (dots = single animal value in each group; Mann-Whitney test; $p > 0.05$).

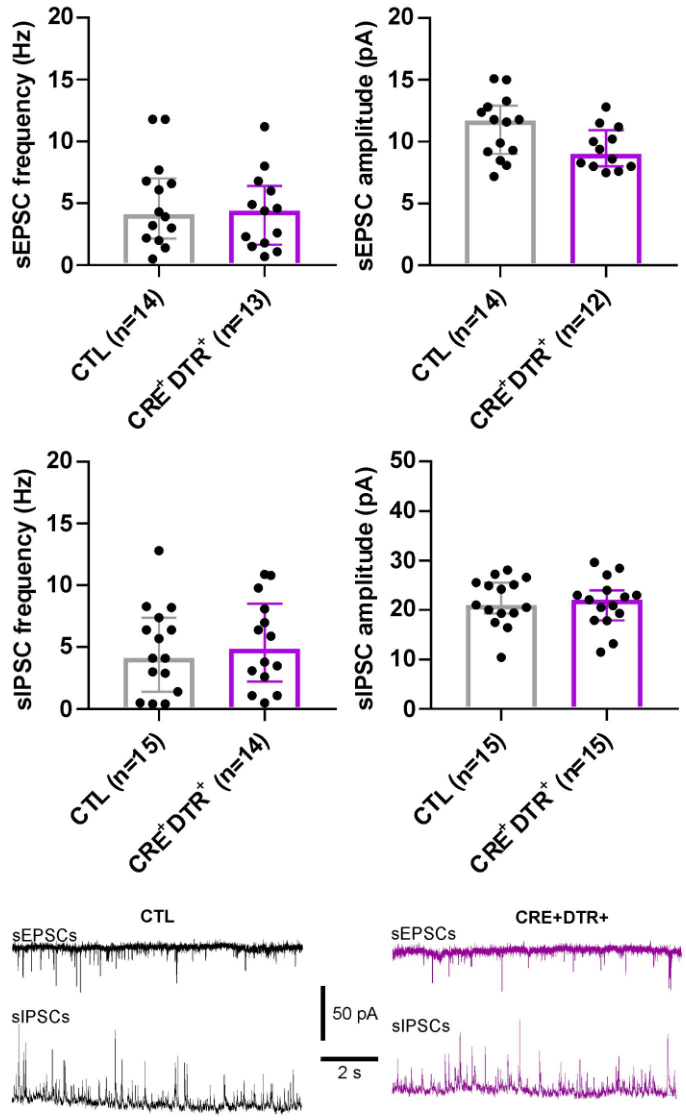

Supplementary Figure S6. The frequency and the amplitude of sEPSCs and sIPSCs was similar between control and CRE+DTR+ mice (Mann-Whitney test; $p > 0.05$;). Traces depict samples of spontaneous activity recorded from a control (black) and a CRE+DTR+ (purple) CPN. Data are presented as median ± interquartile range, dots show single neuron values; n refers to the number of recorded CPNs, which were obtained from 7 CTL and 6 CRE+DTR+ mice.

	CORPUS CALLOSUM				CORTEX			
	Venus+ PDGFRa+ /Venus+	Venus+ Olig2+ /Venus+	CC1+ Venus+ /Venus+	Venus+ PDGFRa+ /PDGFRa+	Venus+ PDGFRa+ /Venus+	Venus+ Olig2+ /Venus+	CC1+ Venus+ /Venus+	Venus+ PDGFRa+ /PDGFRa+
moy	58 %	98 %	18 %	90 %	72 %	98 %	13 %	98 %
sem	*0,9 %*	*1,4 %*	*0,9 %*	*3,2 %*	*8,4 %*	*0,4 %*	*6,2 %*	*0,8 %*

Supplementary Table S1: Histological characterization of Venus+ cells using oligodendrocyte lineage markers.

5’external probe sequence (598pb):

ACTCAGTGTGGGATGCAGAGTACGCAGCTTTCGCTGACTGCATTCTGATTCCCGACCTCATCGGT
GCCTTTTTTTTTTTTTTCCCTCTGAAGTAGTTCAATGTTTTTTTCTTTTGTTTTAAACTGAAGGGTGGA
CCACCAATACTAGCTGGGAACTTAGACGGTAGTAAAAGGGGATAAATGTAAGCCACGGACTTGG
AGAGTCTCTTTCTTCTACTTCAGTTCCCTTCCTTCCGGAAGGTAGAGAAGCTAGAGGCATGAAGTA
GAGCAGGGGTGCCCAGGGGCAGGGCAAACAAAGGGGCAAGGTGGGCACTCCAGACCCCGAAT
CACAGAGCTTGGTCCAGCCTTTGGTTCTGGCGGCTGCGGTCGGTGCTGGCTGGCCTGCAGGAGG
CGCTGTGGCTCGCCTGTAACCCTAAACCTGGCCCCCTCCCTCCACCCTGCCCAGTCTCCTCTCAGG
TCGCAGTTGAAAACAATGCAAACGCTGAGCATAGAGCCTGGGGCGCGGCGGCCTCCGCAGTCCT
GAGCTGGAAGGAAAGGGACTTGGGCTTGGGCCTGGGATCGTCGGGTAGATCCGTGTCGCCTGG
TATCTGGTGGCGATCT

3’ external probe (752 pb):

TGGTGGTGAAGCTAACGATGATAATGATACTAACAGAACACTTTTTCCCCCCTGTATATGTGTATA
AAAATGAAATGAAACTCTAATAGATTCTTCTCTCTCTCTTTAAAATCACAGACCCAGACATGGCCTT
TGTGCCTCTCGGGATGACCGATTCCTTAGTCATCGTGGAAGAGGATGACTCTGCCATCATACCTTG
CCGCACCACAGATCCGGAGACTCAAGTAACCTTGCACAATAACGGGAGGCTGGTGCCTGCCTCCT
ATGACAGCAGGCAGGGCTTCAACGGAACCTTCAGCGTGGGGCCTTACATCTGTGAGGCCACCGT
CAAAGGGAGGACGTTCAAGACCAGCGAGTTTAATGTTTATGCCTTGAAAGGTACTTTCACCTCTCT
AAGTGAGAGCAACAGGCAAACAAGTTTTTTTTTTAATTTGGGTGTTGCCTCTTCCTTCTCTATACAA
CGTGGCTGACCTCTGTAACTATAATAATACAGGCTTAAAAAAATACTACAGGCTGAACTTGCTTCT
ACGTGTAAGACAGCATGTTCCCTGAAAAATACCTCAAGGAATTCTTTCTTGCATAAGCCTCTTACT
ATTTGGTTCCTATTCTTGAATATGCAGCCCTAAGAAGCAAACACACCCCAGGGGGTTGGAGGTCT
AGATTAATTTCAGTAACTTAAGAATCAAGTGATTTCAGTTTTGAAGGAAGACTATAGTTAAAGAG
GCTAAAACTTCTTCCCTTTGATGTGAGGG

Supplementary Table S2: External probes sequences for southern blot.

Oligonucleotides	Sequence	Band size	PCR
PDGFRa5' F<br>PDGFRa5'R	AGACGTCGGGTTTACGAGCAGCGAGAACAGAGTGGC<br>CTCGAGAGCTCCTGAGACCTGCAAATGACAAAAGCC	5,5 kb	95°C 1 min30sec<br>(95°C 20s/58°C 30s/65°C 5min) x 40<br>65°C 10 min
PDGFRa5' F<br>Venus R	GCAATGGAAATAGTCCTTTCTAACC<br>GAAGAAGTCGTGCTGCTTCATGTG	5,2 kb	
Neo F<br>PDGFRa3' R	GGCAGGATCTCCTGTCATCT<br>CCCTCACATCAAAGGGAAGA	5,8 kb	
CRE F<br>CRE R	ATTTGCCTGCATTACCGG<br>ATCAACGTTTTCTTTTCG	350 pb	94°C 1 min<br>(94°C 30s/ 53°C 30s/ 72°C 60s) x 36<br>72°C 10 min
Venus F<br>Venus R	AAAGCTTCATAACTTCGTATAATGTATGCTATACGAAGT<br>TATCCACCATGGTGAGCAAGGGCG<br>GTCGTCCTTGAAGAAGATGGTGCG	350 pb	94°C 2 min<br>(94°C 30s/ 58°C 30s/ 72°C 25s) x 30<br>72°C 10 min
Flipase F<br>Flipase R	CACTGATATTGTAAGTAGTTTGC<br>CTAGTGCGAAGTAGTGATCAGG	725 pb	94°C 90s<br>(94°C 30s/ 58°C 60s/ 72°C 60s) x 35<br>72°C 3 min
Neo F<br>Neo R	GCCCTGAATGAACTGCAGGACG<br>CACGGGTAGCCAACGCTATGTC	514 pb	95°C 3 min<br>(95°C 30s/ 62°C 30s/ 72°C 30s) x 30<br>72°C 5 min
DTR F<br>DTR R	CTTTCTGGCTGCAGTTCTCTC<br>CTCCTTCACATATTTGCATTCTCC	340 pb	94°C 1 min<br>(94°C 30s/ 60°C 30s/ 72°C 25s) x 35<br>72°C 10 min

Supplementary Table S3: Genotyping transgenic mice: primer sequences and PCR protocols.
